# Osteoarthritis in the Middle-Aged and Elderly in China: Prevalence and Influencing Factors

**DOI:** 10.3390/ijerph16234701

**Published:** 2019-11-26

**Authors:** Xueshan Sun, Xuemei Zhen, Xiaoqian Hu, Yuanyuan Li, Shuyan Gu, Yuxuan Gu, Hengjin Dong

**Affiliations:** 1 Center for Health Policy Studies, School of Public Health, Zhejiang University School of Medicine, Hangzhou 310058, Zhejiang, China; sunxueshan@zju.edu.cn (X.S.); zhenxuemei@zju.edu.cn (X.Z.); huxiaoqian@zju.edu.cn (X.H.); sun89521@126.com (Y.L.); guyuxuan@zju.edu.cn (Y.G.); 2 Center for Health Policy and Management Studies, Nanjing University, Nanjing 210093, Jiangsu, China; gushuyan@nju.edu.cn

**Keywords:** osteoarthritis, prevalence, influencing factors, systematic review, meta-analysis, China

## Abstract

*Background*: Osteoarthritis is a common joint disease, with the acceleration of the aging process in China, it has troubled the middle-aged and elderly. There have been some epidemiological studies of osteoarthritis conducted in one single site, and most of them were on knee osteoarthritis. The results varied greatly between different surveys. There was still a lack of large-scale and multicenter epidemiological studies of osteoarthritis. This paper aimed to estimate the overall prevalence of lumbar osteoarthritis, cervical osteoarthritis, hand osteoarthritis, knee osteoarthritis, and hip osteoarthritis in the middle-aged and elderly in China by summarizing the existing publications. *Methods*: We comprehensively searched publications on 1 January 2019 in PubMed, Web of Science, Embase, Cochrane Library, CBM, CNNI, VIP, and Wan Fang. Epidemiological publications on osteoarthritis in the middle-aged and elderly Chinese published from 2000 to 2018 were summarized and analyzed by means of systematic review and meta-analysis. Data of prevalence of osteoarthritis in five joints were extracted from the included publications. The Hoy 2012 tool was used to assess the risk of bias of included studies. *Results*: After performing a systematic search in eight databases and manually searching, 3058 articles were obtained, and 21 articles were included in the meta-analysis. Lumbar osteoarthritis was the most prevalent with a prevalence of 25.03% (95% CI: 0.1444–0.3562). The prevalence of knee osteoarthritis followed, which was 21.51% (95% CI: 0.1873–0.2429). The prevalence of cervical osteoarthritis was 20.46% (95% CI: 0.1244–0.2849). The prevalence of hand osteoarthritis was 8.99% (95% CI: 0.0435–0.1364). The prevalence of hip osteoarthritis was not pooled due to its lack of data. Higher prevalence of knee, hand, lumbar, and cervical osteoarthritis was seen in the female group and southern regions. The prevalence of knee and hand osteoarthritis increased with age. The prevalence of lumbar and cervical osteoarthritis increased with age. There was also a trend that the prevalence increased with age before 70 years old and slightly decreased in the oldest ages. **Conclusions**: The lumbar joint was the joint most prevalently affected by osteoarthritis, followed by the prevalence of knee, cervical, hand, and hip joint osteoarthritis. Women, the southern population, and the older population are more susceptible to osteoarthritis. The paucity of epidemiology data of osteoarthritis in China appeals for more population-based surveys being conducted in the future. Based on the relatively high prevalence of osteoarthritis obtained from this review, self-management and community-based management should be considered, which can provide experience from the management of hypertensions and diabetes.

## 1. Introduction

Osteoarthritis (OA) is a chronic and degenerative joint disease that is part of the aging process. It is characterized by loss and degradation of articular cartilage in addition to synovial inflammation, leading to joint stiffness, swelling, pain, and loss of mobility [1,2,3]. The cause of the onset of OA is not clear, but some factors are considered to be influencing factors (such as aging, obesity, inflammation, trauma, joint overuse, metabolic disorders, heredity, and so on.) [3,4]. The treatment of OA has been a kind of staged treatment, and mainly a nonoperative treatment, such as physical activity, nutrition, and drug treatment [5]. OA usually occurs in knee, lumbar, cervical, hand, and hip joints, and because of the higher knee’s vulnerability to direct (knocked) and indirect (twisted) trauma, along with the high load supported by this joint, the knee is seen as the most frequently affected joint by OA [1], and also the most studied joint.

OA is a major public health problem [6]. It has been listed as the fastest increasing major health condition and ranked second as a cause of disability by the World Health Organization (WHO) [7]. The WHO Scientific Group on Rheumatic Diseases estimates that 10% of the world’s population who are 60 years or older have significant clinical problems that can be attributed to OA [8]. Because the burden of osteoarthritis has been heavier and heavier, prevention, treatments, and management for OA have been the focus of research on OA. Internationally, in 2013, American Academy of Orthopaedic Surgeons (AAOS) published a treatment guideline for knee OA to provide the recommendations based on the results of evidence-based medicine [8]. In 2012, the OA Research Society International (OARSI) published an update to their evidence-based, consensus recommendations for the treatment of OA of the hip and knee joints [9]. In China, the Chinese Medical Association Orthopaedic Society released the guidelines for diagnosis and treatment of osteoarthritis in 2018, providing great recommendations [10].

The epidemiological character of OA in China can play a vital role in making policy for diagnosis, treatment, and management, which is also the precondition when making policy. The prevalence of osteoarthritis in different joints is consistently seen to increase with age [11], and with the acceleration of China’s aging process and longer life expectancy, the prevalence of OA may increase significantly in the future. Only by having an exact knowledge of the number of patients and the distribution, the policy could be made more scientific. While in China, there are very limited data of large, multicenter, epidemic research on the prevalence of OA [12]. Previous research has been conducted in the scale of one province or one city. Furthermore, huge differences between the current studies can be found due to the difference in mean age, geographic regions, ratio of sex, diagnosis criteria, and so on. For example, Wang et al. conducted an epidemic research study in Xi’an City (northern city) in 2007 and they reported a 9.5% prevalence of knee osteoarthritis among people aged 40 years old and over [13]. Meanwhile, Ren ZhiJian conducted research in Wenzhou City, Zhejiang Province in 2017, and the prevalence of knee osteoarthritis they reported was 39.11% (southern city) [14]. The overall prevalence of osteoarthritis is still not clear, which causes great confusion when making some strategies for OA patients. In addition to the population-based survey, there was a review conducted by Tie (published in a Chinese journal) to summarize the prevalence of knee OA, based on the existing articles, which reported that the prevalence of knee OA was 17% [12]. As we know, there has been no other evidence-based review to report the prevalence of OA in lumbar, cervical, hip, and hand joints in the middle-aged and elderly in China.

This paper aimed to pool OA prevalence data from current publications and to provide accurate estimates of the prevalence of OA in knee, lumbar, cervical, hand, and hip joints. “The middle-aged and elderly” refers to individuals who are aged 40 years old and over. It may be the first systematic review to provide overall estimates for the prevalence of osteoarthritis in the middle-aged and elderly in China, hoping to fill the vacancy and provide evidence for policy makers.

## 2. Methods

### 2.1. Search Strategy

Eight databases were searched for publications. Four Chinese databases (CBM, CNNI, VIP, and Wan Fang) and four foreign databases (Pubmed, Web of Science, Cochrane Library, and Embase) were searched between 1 January 2000 and 31 December 2018 for epidemiological studies investigating the prevalence of OA, with a language restriction of English or Chinese. The key words for searching were “prevalence”, “osteoarthritis”, and “China”. A detailed search strategy can be found in Appendix A. We also screened the references of included studies. Two reviewers (X.S. and X.M.) reviewed titles and abstracts. After screening, full texts were read. When disagreements between two reviewers occurred, a third reviewer (H.J.) would join in the discussion and made a final decision with the two reviewers together.

### 2.2. Studies Selection Criteria

All published studies investigating the prevalence of OA or its subtypes were considered in this review. The inclusion and exclusion criteria are as follows. Studies would be included when they satisfied these criteria: (1) The study was an epidemiological survey of OA (or its subtypes) and reported prevalence data; (2) the study reported diagnosis criteria for OA; (3) the study conformed to the search strategy; (4) the study was based on the general population. Studies would be excluded if (1) the study did not conform to the search strategy; (2) the study focused on people with particular occupations or was based on patients in hospitals; (3) the study was a review or an abstract; (4) the study was not on humans.

### 2.3. Data Extraction and Synthesis

A standardized form was designed and used by the reviewers, and they independently extracted descriptive information of the studies, including authors, published year, research regions, sampling method, diagnosis criteria, subtypes of OA, age, and sex. In order to pool the overall prevalence of OA, the prevalence data were extracted, including the number of samples and number of OA patients in each age group. If the study reported the number of sample and the prevalence of osteoarthritis, the number of OA patients would be calculated. Data were synthesized using Stata 14.0, and because the prevalence of OA was not expected to be closer to 0 or 1, a nontransformation method was used. Standard errors (SEs) for prevalence (p) estimates were derived from the equation $\sqrt{\left[\left(p\times \left(1-p\right)\right)/n\right]}\;$, where *n* = number of participants with completed data in the publications [15,16].

Heterogeneity was assessed using the method of Q-test. If I-square was greater than or equal to 50% [7,17], the heterogeneity was supposed statistically significant. For pooling, a random-effects model was used when heterogeneity was identified and a fixed-effects model was used when heterogeneity was not identified [1].

### 2.4. Quality of Studies

The risk of bias of each study included in this review was appraised by the Hoy 2012 tool [18], which is a tool developed specifically for prevalence studies [15]. Ten items were included in the Hoy 2012 tool, where each item was assessed as low risk, high risk, and unclear risk of bias. Unclear risk of bias was regarded as high risk of bias [7]. The items included: Representation, sampling, random selection, nonresponse bias, data collection, case definition, reliability and validity of study tool, data collection, prevalence period, numerators, and denominators [18]. Finally, the number of high risk and unclear answers were counted as an overall score. If the score was less than or equal to 2, the risk was low; if the score was between 3 and 4, the risk was moderate; if the score was higher than or equal to 5, the risk was high [7,18].

## 3. Results

### 3.1. Literature Search

**Figure 1:**
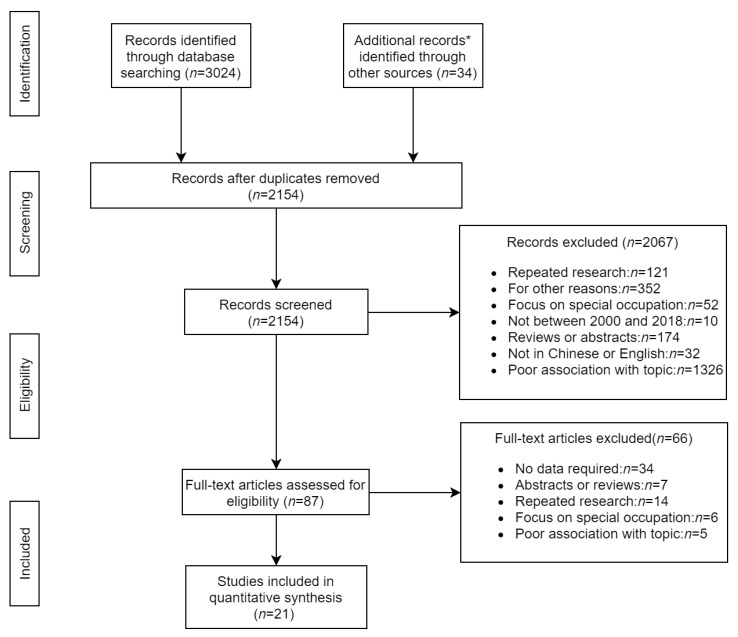
The flowchart of search results. * Additional studies are studies that were searched through manual search; OA: Osteoarthritis.

After searching eight databases, 3024 papers were obtained (Embase: 689, Pubmed: 516, Web of science: 313, Cochrane Library: 34, CNKI: 199, Wan Fang: 567, VIP: 396, CBM: 310), and with 34 additional papers obtained by hand searching (the detailed search strategies can be found in Appendix A). After removing duplicates, 904 papers were excluded. In the remaining 2154 papers, we screened the titles and abstracts. After screening, 2067 papers were excluded because they did not meet the study selection criteria or for other reasons. We read full-texts of 87 papers, and 33 of them did not have detailed prevalence data for osteoarthritis, 7 of them were abstracts, 14 of them were repeated research, 6 of them were focused on a particular occupation or were based on patients in hospitals, and 5 of them had poor association with our topic (not about osteoarthritis or the prevalence of osteoarthritis). Finally, 21 papers were included in the quantitative synthesis (Figure 1).

### 3.2. Characteristics of Included Publications

In the 21 included studies, 20 studies reported the prevalence of knee OA [13,14,19,20,21,22,23,24,25,26,27,28,29,30,31,32,33,34,35,36,37]. Six studies reported the prevalence of lumbar and cervical OA [13,32,34,35,36], seven studies reported the prevalence of hand OA [13,32,33,34,35,36,37], and one study reported the prevalence of hip OA [37]. Of the included studies, 14 of them did research on people 40 years old and above, four studies focused on people 50 years old and above, three studies focused on 60-year-olds and above. Not all included studies used the same criteria to diagnosis OA, as three studies used “of the Research Group of Beijing Hospital, Ministry of Health”, four studies used “Framingham Standards”, four studies used “Standards of Chinese Medical Association”, and 10 studies used “ACR osteoarthritis criteria”. The sample size ranged from 202 to 6218, and the publication year ranged from 2005 to 2017. Twenty were single-site studies and the study conducted by Xue Qingyun was a multisite study (Table 1).

### 3.3. Risk of Bias

By using the Hoy 2012 tool, the risk of bias for each study was assessed. From the result, it could be found that 16 studies had a score of “1”, which means the risks of bias were low, and five studies had a score of “2”, which also means the risks of bias were low. It should be focused on that the first parameter “Was the study population a close representation of the national population?” achieved 20 “N” out of 21 studies; because 20 studies were single-site studies. According to the results, all included studies reported a low risk of bias through assessing (Table 2).

### 3.4. Prevalence of Osteoarthritis

**Figure 2:**
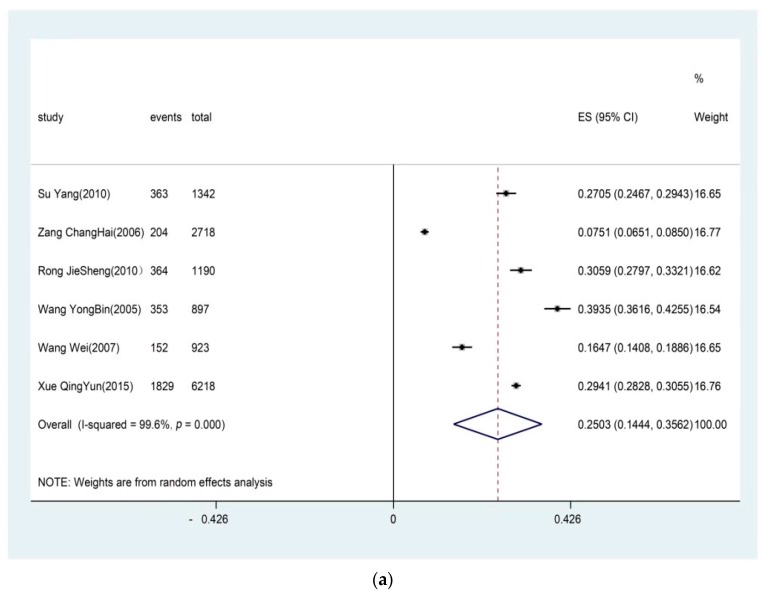

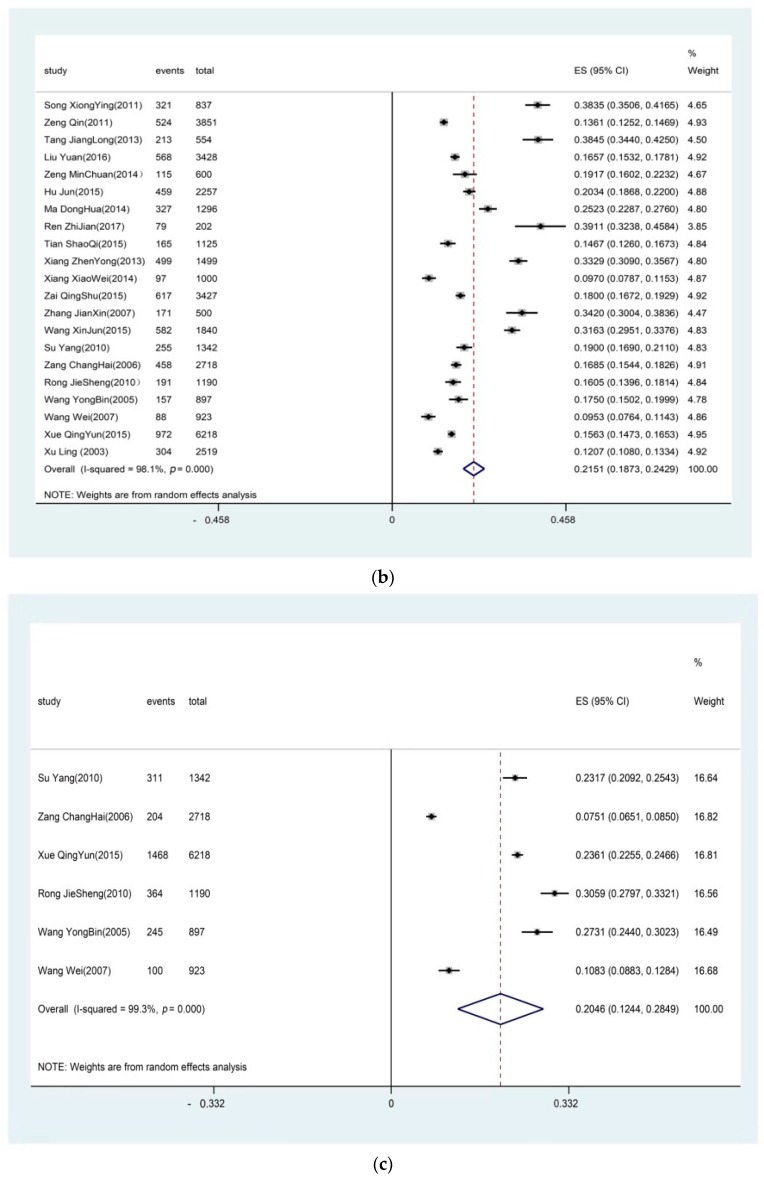

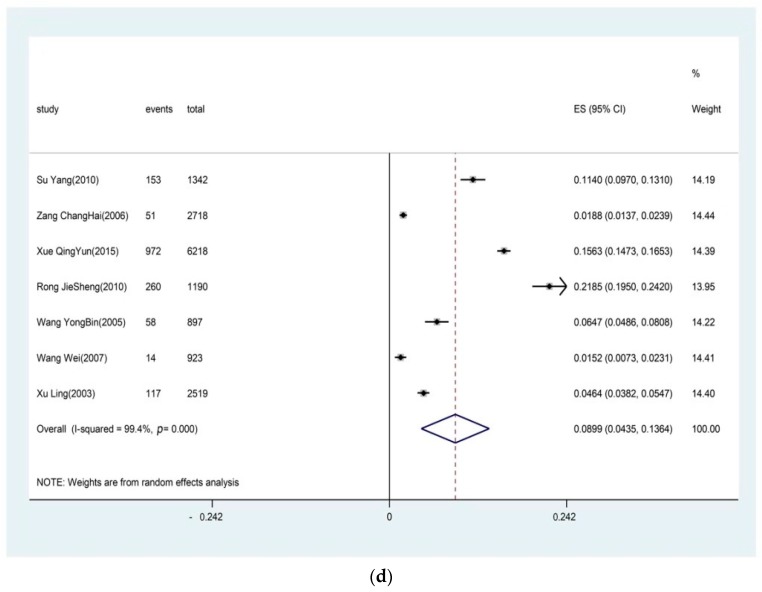
Pooled estimate for the prevalence of (**a**) lumbar OA; (**b**) knee OA; (**c**) cervical OA; (**d**) hand OA.

Six studies reported the prevalence of lumbar OA. The prevalence of lumbar OA ranged from 7.51% [33] to 39.35% [35]. Pooling the results from the six studies, the overall prevalence of lumbar OA was 25.03% (95% CI: 0.1444–0.3562, I^2^ = 99.66%, *p* < 0.0001, Figure 2a) by using a random effects model. Twenty-one studies reported the prevalence of knee OA, the prevalence varied from 9.5% to 38.45%. The pooled estimate for knee OA prevalence was 21.51% (95% CI: 0.1873–0.2429, I^2^ = 98.1%, *p* < 0.0001, Figure 2b) by using a random effects model. Six studies reported the prevalence of cervical OA. The prevalence of cervical OA ranged from 7.51% [33] to 30.99% [34]. Pooling the result of the six studies, the prevalence of cervical OA was 20.46% (95% CI: 0.1244–0.2849, I^2^ = 99.3%, *p* < 0.0001, Figure 2c) by using a random effects model. Seven studies reported the prevalence of hand OA. The prevalence of hand OA ranged from 1.52% [13] to 21.85% [34]. Pooling the results from the seven studies, the overall prevalence of hand OA was 8.99% (95% CI: 0.0435–0.1364, I^2^ = 99.4%, *p* < 0.0001, Figure 2d) by using a random effects model. There was one paper included in the analysis of the prevalence of hip OA. Xu et al. conducted an epidemiological research study in Beijing, comparing the prevalence of knee osteoarthritis, hip osteoarthritis, and hand osteoarthritis in the middle-aged and elderly in Beijing with that of American White [37] in 2003. According to the research, there was no case of clinical hip OA in the male group, but there was one case in the female group (combing the results of radiological diagnosis and symptom), where the sample size was 1000 male and 1500 female. Hip OA was rare in Beijing, China [37].

### 3.5. Influencing Factors for the Prevalence of Osteoarthritis

Subgroup analysis was conducted for knee OA, lumbar OA, cervical OA, and hand OA to explore the influencing factors for the prevalence and the source of heterogeneity (Table 3). For knee OA, there were 13 publications, 21 publications, 21 publications, and 17 publications reporting prevalence by sex, regions, diagnostic criteria, and age, respectively. For lumbar OA, there were four publications, six publications, six publications, and six publications reporting prevalence by sex, regions, diagnostic criteria, and age, respectively. For cervical OA, there were four publications, six publications, six publications, and six publications reported prevalence by sex, regions, diagnostic criteria, and age, respectively. For hand OA, there were five publications, seven publications, seven publications, and six publications reported prevalence by sex, regions, diagnostic criteria, and age, respectively. It indicated that sex, age, diagnostic criteria, and geographical region were not the source of heterogeneity. Different diagnostic criteria for OA were categorized into four main categories: (1) Beijing Hospitals—Diagnostic criteria for OA of the Research Group of Beijing Hospital, Ministry of Health; (2) Chinese Medical Association—Chinese Medical Association diagnostic criteria for OA, 2007; (3) Framingham—Framingham diagnostic criteria for OA; (4) ACR—ACR diagnostic criteria for OA (includes different versions and criteria for different joints).

As for sex, it can be found that the prevalence of OA in females was higher than in males in lumbar, knee, cervical, and hand joints. The highest difference of prevalence between the two sexes was in the knee joint, where the prevalence for females was 25.55% and the prevalence for males was 14.20%. As for age, there was an obvious trend that the prevalence of OA increased with age, although a slightly higher prevalence was found for the 60–69-years-old group than for the 70-years-old-and-above group in cervical OA and lumbar OA. As for geographic regions, it indicated that the prevalence of OA was higher in southern China than that in northern China. As for diagnostic criteria, a huge difference can be found between different criteria, such as the prevalence of knee OA by using the “Framingham diagnostic criteria for OA”, which was 14.81%, while it was 30.09% by using the “Chinese Medical Association diagnostic criteria for OA, 2007” criteria. For OA in four joints, the OA prevalence of people in each group of the four categories was all statistically significant (Table 3).

## 4. Discussion

This is the first systematic review and meta-analysis to provide the overall prevalence of OA in the middle-aged and elderly in China. It presented pooled population-based prevalence of OA in knee, lumbar, cervical, and hand joints. For hip OA, there were not enough articles available to conduct a meta-analysis; therefore, the prevalence of hip OA was just displayed. In addition, the distribution of prevalence of OA between age, sex, region, and diagnostic criteria was obtained by subgroup analysis. Before this review, the overall prevalence of OA in the middle-aged and elderly in China stayed unknown. There is a lack of larger-scale and multicenter epidemiological research for the prevalence of OA in China, which can also be concluded by systematic researching. Twenty-one articles were included in this paper, 20 of which were conducted in the scale of one province or city, and only one article conducted research in six cities of China. In addition, the prevalence of OA reported in different research studies had a huge difference to each other, making it unclear how to outline the overall prevalence according to an individual research study. Lastly, there were more research studies focused on the prevalence of knee OA, while sparse studies were conducted for lumbar OA, cervical OA, and hand OA. By systematic review, 21 articles reported the prevalence of knee OA, with 6–7 articles reporting the prevalence of lumbar OA, cervical OA, and hand OA and one article reporting the prevalence of hip OA. This review provided data on the overall prevalence of OA by pooling existing articles, filling the vacancies in the field of epidemic research for OA in China.

It can be concluded from the results that lumbar OA was the most prevalent, followed by the prevalence of knee OA, cervical OA, and hand OA. Hip OA was rare in China [37]. It was supposed that the knee should be the most prevalently affected joint, given the heavy burden that knee joints bear; however, according to the results, lumbar OA was the most prevalent among the five joints. The result that lumbar OA was the most prevalent was consistent with Wang and Xue’s findings [35]. Maybe because of the high level of mobility and load forces, the facet joints can be a potentially important source of symptoms, especially in the lumbar area [38]. According to Xue’s research, the common risk factors of lumbar OA in the six cities are age and the Chinese life style of squatting defecation, which may explain the relatively higher prevalence of lumbar OA [39]. The high prevalence of lumbar OA should be paid great attention because the osteoarthritic (facet joint changes) and degenerative alternation (disk area changes) in lumbar joints can be potential sources of low back pain (LBP), which is widespread and is the second most common concern expressed by patients in primary care [40].

Through the subgroup analysis of the prevalence of OA in sex, age, regions, and diagnosis criteria, the distributions of OA can be found. A trend can be seen that the prevalence of OA in females, southern regions, and the elderly was higher, which was consistent with the findings of Felson et al. [41]. They conducted the Framingham Osteoarthritis Study and found that the prevalence increased with age. They supposed that aging cartilage (shorter proteome chains, decreased water content) may be susceptible to fatigue fractures, producing OA; the increased subchondral stiffness to trabecular microfractures may account for increased OA (this increased bony stiffness remains to be identified); and that neuromuscular changes with age may predispose the joint to damages, resulting in OA [41]. The gender-related prevalence finding was consistent with most of the survey [41,42]. The gender-related prevalence may be attributed to estrogen levels and different health-seeking behavior [32,42]. Estrogen levels in postmenopausal women are significantly lower than normal [29]. Because of the difference of health-seeking behavior between females and males, women are more sensitive to the changes of their joints [39]. The result that the prevalence was higher in southern regions was not consistent with Tie’s findings [12]. It was supposed that the difference between southern regions and northern regions was mostly temperature and humidity, and a higher prevalence of OA can occur where it is cold and damp [12]. With the improvement in economic conditions, the heating system in the northern regions has greatly improved and less people work outside than before. The cold weather may not affect people’s joints in the northern regions; however, there are more rains in the southern regions than northern regions, and the southern region is more humid, which may be contributable to the higher prevalence of OA.

Three suggestions based on the findings of this review can be provided for policy makers. First, more epidemiological data on OA should be collected through multiple ways. Through the systematic review, there was no national population-based survey to report the overall prevalence in China, while the survey for lumbar OA, cervical OA, hand OA, and hip OA was especially sparse. In order to have a thorough knowledge of the prevalence of OA in China, providing more evidence for policy makers, more population-based surveys are urgently needed. Meanwhile, one more item about OA could be considered to be added by the Chinese National Health Service Survey, which is conducted every five years. The question can be on the self-reported condition of OA, to obtain the epidemiological character for OA in the whole countryside from the view of self-reported prevalence. Furthermore, to solve the lack of data of lumbar OA, cervical OA, hand OA, and hip OA, more joints can be considered for inclusion in the surveys conducted in the future to provide more evidence for other joints. Based on the results, the prevalence of OA in several joints was relatively high and increased with age. With the aging process, the prevalence of OA will continue to increase, and the number of OA patients will increase year by year. To add, the WHO estimated that the OA would be the main crippling disease, causing great disease burden for society. According to the Fifth Chinese National Health Service Survey [43], the prevalence of hypertension and diabetes was lower than the prevalence of OA in this review. Both hypertension and diabetes have been included in Key Chronic Disease Management in China; however, the management of OA has not been heightened. There are clear targets and end points to treating hypertension and diabetes, the goal of treating OA being pain/symptom relief. Compared with using healthcare in hospitals, more management and treatment of OA can be undertaken by primary healthcare institutions and communities. It can draw experience from the management of hypertension and diabetes, making full use of community medical care and primary medical care to carry out community-based health management for OA [44,45]. Meanwhile, more advertising about the prevention, diagnosis, and treatment of OA can be carried out in the communities to improve the initiative of self-management among the middle-aged and elderly population. Last, but not the least, due to the lack of a population-based survey, the method evidence-based medicine shows great advantages in obtaining the overall prevalence of OA.

To our knowledge, this is the first systematic review to explore the overall prevalence of OA in lumbar, knee, cervical, hand, and hip joints in China. However, there are still some limitations in this study. First, the heterogeneity between different studies was statistically significant; therefore, the results should be adopted with caution. To solve this, a random effects model was adopted when the heterogeneity was significant. Second, due to different diagnosis criteria adopted in the included articles, the prevalence extracted from the papers was not calculated by the same criteria, which may affect the polled results. To solve this, subgroup analysis was conducted for the articles with different diagnosis criteria. Third, compared with knee OA, the small number of articles included for lumbar OA, cervical OA, and hand OA may have caused the pooled estimates to be unstable. With more research conducted in the future, these results may be affected.

## 5. Conclusions

This is the first systematic review to provide pooled prevalence of OA by summarizing the existed publications. Although knee OA was the most studied subtype, it was seen that lumbar OA was the most prevalent among the five joints. It showed a trend that the prevalence of OA was higher in the female population, southern regions, and the older population. The number of OA patients will likely become more and more in China with the rapid aging process, so more actions should be taken to attach great importance to OA. Community-based management and self-management for OA should be promoted based on full use of the primary healthcare, and the epidemiological character of OA should be further explored by the Chinese National Health Survey and province-based surveys in the future.

## Figures and Tables

**Table 1:** Characteristics of included studies.

Study ID	Regions	Sample (Male)	Sampling	Diseased Joints	Diagnosis Criteria	Age Setting
Song XiongYing (2011) [19]	Beijing	837 (266)	Hierarchical cluster random sampling	Knee	Criteria for knee osteoarthritis of the Research Group of Beijing Hospital, Ministry of Health	≥40
Zeng Qin (2011) [20]	Guangzhou	3851 (1845)	Stratified random sampling	Knee	Framingham diagnostic criteria for OA	≥50
Tang JiangLong (2013) [21]	Yunnan	554 (252)	Cluster random sampling	Knee	Chinese Medical Association diagnostic criteria for knee OA, 2007	≥60
Liu Yuan (2016) [22]	Jiangsu	3428 (1661)	Stratified random sampling	Knee	Framingham diagnostic criteria for knee OA	≥40
Zeng MinChuan (2014) [23]	Hunan	600 (262)	Hierarchical cluster random sampling	Knee	ACR diagnostic criteria for knee OA, 1995	≥40
Hu Jun (2015) [24]	Jiangsu	2257 (1221)	Simple random sampling	Knee	ACR diagnostic criteria for knee OA, 1986	≥50
Ma DongHua (2014) [25]	Hunan	1296 (533)	Unclear	Knee	Chinese Medical Association diagnostic criteria for knee OA, 2007	≥40
Ren ZhiJian (2017) [14]	Zhejiang	202 (113)	Simple random sampling	Knee	Chinese Medical Association diagnostic criteria for knee OA, 2007	≥40
Tian ShaoQi (2015) [26]	Shandong	1125 (540)	Hierarchical cluster random sampling	Knee	ACR diagnostic criteria for knee OA, 1995	≥40
Xiang ZhenYong (2013) [27]	Shanghai	1499 (709)	Hierarchical cluster random sampling	Knee	ACR diagnostic criteria for knee OA, 2000	≥40
Xiang XiaoWei (2014) [28]	Shenzhen	1000 (500)	Simple random sampling	Knee	ACR diagnostic criteria for knee OA, 1995	≥50
ZaiQingShu (2015) [29]	Shandong	3427 (617)	Simple random sampling	Knee	ACR diagnostic criteria for knee OA, 1995	≥40
Zhang JianXin (2007) [30]	Fujian	500 (198)	Cluster random sampling	Knee	ACR diagnostic criteria for knee OA, 1995	≥60
Wang XinJun (2015) [31]	Xinjiang	1840 (857)	Cluster random sampling	Knee	ACR diagnostic criteria for knee OA, not mentioned version	≥50
SuYang (2010) [32]	Guangzhou	1342 (640)	Hierarchical cluster random sampling	Knee, lumbar, cervical, hand	Chinese Medical Association diagnostic criteria for knee OA, 2007	≥40
Zang ChangHai (2006) [33]	Shanxi	2718 (1858)	Cluster random sampling	Knee, lumbar, cervical, hand	ACR diagnostic criteria for knee OA, 1986; ACR diagnostic criteria for hand OA, 1990	≥40
Rong JieSheng (2010) [34]	Heilongjiang	1190 (573)	Hierarchical cluster random sampling	Knee, lumbar, cervical, hand	Diagnostic criteria for knee osteoarthritis of the Research Group of Beijing Hospital, Ministry of Health	≥40
Wang YongBin (2005) [35]	Shanghai	897 (377)	Cluster random sampling	Knee, lumbar, cervical, hand	Framingham diagnostic criteria for OA	≥40
Wang Wei (2007) [13]	Xi’an	923 (448)	Hierarchical cluster random sampling	Knee, lumbar, cervical, hand	Criteria for knee osteoarthritis of the Research Group of Beijing Hospital, Ministry of Health	≥40
XueQingYun (2015) [36]	Six Cities	6218 (3302)	Cluster random sampling	Knee, lumbar, cervical, hand	ACR diagnostic criteria for knee OA, 1995	≥40
Xu Ling (2003) [37]	Beijing	2519 (1012)	Stratified random sampling	Knee, hip, hand	Framingham diagnostic criteria for OA	≥60

**Table 2:** Risk of bias assessment of included studies using Hoy 2012 tool.

	Score	1	2	3	4	5	6	7	8	9	10	Score
Study ID												
Song XiongYing (2011) [19]		N	Y	Y	Y	Y	Y	Y	Y	Y	Y	1
Zeng Qin (2011) [20]		N	Y	Y	Y	Y	Y	Y	N	Y	Y	2
Tang JiangLong (2013) [21]		N	Y	Y	Y	Y	Y	Y	Y	Y	Y	1
Liu Yuan (2016) [22]		N	Y	Y	Y	Y	N	Y	Y	Y	Y	2
Zeng MinChuan (2014) [23]		N	Y	Y	Y	Y	Y	Y	Y	Y	Y	1
Hu Jun (2015) [24]		N	Y	Y	Y	Y	Y	Y	Y	Y	Y	1
Ma DongHua (2014) [25]		N	Y	N	Y	Y	Y	Y	Y	Y	Y	2
Ren ZhiJian (2017) [14]		N	Y	Y	Y	Y	Y	Y	Y	Y	Y	1
Tian ShaoQi (2015) [26]		N	Y	Y	Y	Y	Y	Y	Y	Y	Y	1
Xiang ZhenYong (2013) [27]		N	Y	Y	Y	Y	Y	Y	Y	Y	Y	1
Xiang XiaoWei (2014) [28]		N	Y	Y	Y	Y	Y	Y	Y	Y	Y	1
ZaiQingShu (2015) [29]		N	Y	Y	Y	Y	Y	Y	Y	Y	Y	1
Zhang JianXin (2007) [30]		N	U	Y	Y	Y	Y	Y	Y	Y	Y	2
Wang XinJun (2015) [31]		N	Y	Y	Y	Y	Y	Y	Y	Y	Y	1
SuYang (2010) [32]		N	Y	Y	Y	Y	Y	Y	Y	Y	Y	1
Zang ChangHai (2006) [33]		N	Y	Y	Y	Y	Y	Y	Y	Y	Y	1
Rong JieSheng (2010) [34]		N	Y	Y	Y	Y	Y	Y	Y	Y	Y	1
Wang YongBin (2005) [35]		N	N	Y	Y	Y	Y	Y	Y	Y	Y	2
Wang Wei (2007) [13]		N	Y	Y	Y	Y	Y	Y	Y	Y	Y	1
XueQingYun (2015) [36]		Y	U	Y	Y	Y	Y	Y	Y	Y	Y	1
Xu Ling (2003) [37]		N	Y	Y	Y	Y	Y	Y	Y	Y	Y	1

Note: Y = Yes (low risk), N = No (high risk), U = Unclear, Score = the number of “N”. 1: Was the study population a close representation of the national population?; 2: Was the sampling frame a true or close representation of the target population?; 3: Was some form of random selection used to select the sample OR was a census undertaken?; 4: Was the likelihood of nonresponse bias minimal?; 5: Were data collected directly from the subject?; 6: Was an acceptable case definition used in the study?; 7: Was the study instrument that measured the parameter of interest shown to have reliability and validity?; 8: Was the same mode of data collection used for all subjects?; 9: Was the length of the prevalence period for the parameter of interest appropriate?; 10: Were the numerator(s) and denominator(s) for the parameter of interest appropriate?

**Table 3:** Subgroup analysis of the prevalence of OA.

Influencing Factors	Knee OA		Lumbar OA		Cervical OA		Hand OA	
	Prevalence (%) (95% CI)	I^2^ (%)	Prevalence (%) (95% CI)	I^2^ (%)	Prevalence (%) (95% CI)	I^2^ (%)	Prevalence (%) (95% CI)	I^2^ (%)
Sex	*p*-value < 0.001		*p*-value < 0.001		*p*-value < 0.001		*p*-value < 0.001	
Male	14.20 (11.26–17.13)	94.7	18.32 (6.13–30.52)	98.8	13.63 (4.24–23.02)	98.5	5.39 (2.49–8.28)	97.4
Female	25.55 (20.35–30.76)	98.0	22.13 (10.18–34.08)	98.6	20.91 (7.64–34.17)	99.1	5.66 (2.20–9.11)	97.2
Age	*p*-value < 0.001		*p*-value < 0.001		*p*-value < 0.001		*p*-value < 0.001	
40–49	8.24 (06.12–10.36)	93.7	19.07 (7.55–30.60)	99.2	11.44 (7.38–15.49)	95.2	3.59 (1.37–5.82)	94.0
50–59	18.42 (14.34–22..51)	97.4	29.76 (14.38–45.13)	99.2	23.51 (14.57–32.45)	98.0	7.83 (4.06–11.6)	97.2
60–69	23.97 (19.61–28.33)	95.6	39.71 (19.97–59.45)	98.9	27.42 (17.51–37.32)	96.2	14.11 (7.05–21.18)	96.8
70–	31.27 (25.58–36.96)	95.0	37.80 (18.90–56.70)	98.2	26.83 (12.10–41.56)	98.2	15.75 (7.74–23.76)	96.4
Standards	*p*-value < 0.001		*p*-value < 0.001		*p*-value < 0.001		*p*-value < 0.001	
Beijing Hospitals	21.25 (7.11–35.38)	99.1	23.52 (9.68–37.36)	98.4	23.17 (20.92–25.43)	-	11.65 (−8.27–31.58)	99.6
Framingham	14.81 (12.52–17.10)	90.8	39.35 (36.16–42.55)	-	15.56 (−0.22–40.05)	99.8	5.42 (3.65–7.18)	74.4
Chinese Medical Association	30.09 (21.44–38.74)	96.7	27.05 (24.67–29.43)	-	20.69 (1.33–40.05))	99.3	11.40 (9.7–13.10)	-
ACR	21.21 (17.19–25.24)	98.2	18.46 (−3.01–39.93)	99.9	27.31 (24.40–30.23)	-	8.75 (−4.73–22.23)	99.9
Regions	*p*-value < 0.001		*p*-value < 0.001		*p*-value < 0.001		*p*-value < 0.001	
Southern	20.39 (17.66–23.12)	98.0	31.01 (26.95–35.08)	90.6	23.99 (21.21–26.77)	82.8	9.55 (4.92–14.19)	97.6
Northern	17.46 (13.35–21.57)	98.3	23.33 (12.86–33.81)	99.3	19.74 (10.98–28.50)	99.2	6.33 (3.62–9.04)	98.5

Note: *p*-value means the difference of the prevalence of OA in one joint between different groups in the same category (e.g., *p*-value of the prevalence of knee OA between males and females).

## List of Abbreviations

OA	Osteoarthritis
WHO	World Health Organization
AAOS	American Academy of Orthopaedic Surgeons
OARSI	OA Research Society International

## Appendix A. Search Strategies

### Appendix A.1. English Database

#### Appendix A.1.1. Search strategies of Pub Med

**Table A1:** Search strategies of Pub Med

Search	Query	Items Found
#1	Search ((((((((Osteoarthritis [MeSH Terms]) OR osteoarthritis) OR osteoarthrosis) OR osteoarthrosis) OR arthritis, degenerative) OR arthritides, degenerative) OR degenerative arthritides) OR degenerative arthritis) OR osteoarthrosis deformans Sort by: [pubsolr12]	83,796
#2	Search ((((Prevalence rate) OR epidemic) OR epidemiology) OR epidemiological investigation) OR distribution	3,213,937
#3	Search China	1,427,446
#4	Search (((((((((((Osteoarthritis[MeSH Terms]) OR osteoarthritis) OR osteoarthrosis) OR osteoarthrosis) OR arthritis, degenerative) OR arthritides, degenerative) OR degenerative arthritides) OR degenerative arthritis) OR osteoarthrosis deformans)) AND (((((Prevalence rate) OR epidemic) OR epidemiology) OR epidemiological investigation) OR distribution)) AND China	545
#5	Search (((((((((((Osteoarthritis[MeSH Terms]) OR osteoarthritis) OR osteoarthrosis) OR osteoarthrosis) OR arthritis, degenerative) OR arthritides, degenerative) OR degenerative arthritides) OR degenerative arthritis) OR osteoarthrosis deformans)) AND (((((Prevalence rate) OR epidemic) OR epidemiology) OR epidemiological investigation) OR distribution)) AND China Filters: Publication date from 2000/01/01 to 2018/12/31 Sort by: [pubsolr12]	516

#### Appendix A.1.2. Search strategies of Web of Science

**Table A2:** Search strategies of Web of Science

Search	Query	Items Found
#1	TS = osteoarthritis OR TS = osteoarthrosis OR TS = arthritis, degenerative OR TS = arthritis, degenerative OR TS = degenerative arthritis OR TS = degenerative arthritides OR TS = osteoarthritis deformas	156,962
#2	TS = prevalence rate OR TS = epidemic OR TS = epidemiological investigation OR TS = distribution	7,806,811
#3	TS = China	1,123,241
#4	#3 AND #2 AND #1 (Date: 2000–2018)	313

#### Appendix A.1.3. Search strategies of Cochrane Library

**Table A3:** Search strategies of Cochrane Library

Search	Query	Items
#1	MeSH descriptor: [Osteoarthritis] explode all trees	6592
#2	(osteoarthrosis) (Word variations have been searched)	501
#3	(arthritis, degenerative) (Word variations have been searched)	380
#4	(arthritides, degenerative) (Word variations have been searched)	4
#5	(degenerative arthritides) (Word variations have been searched)	4
#6	(degenerative arthritis) (Word variations have been searched)	380
#7	(osteoarthrosis deformans) (Word variations have been searched)	7
#8	(Prevalence rate) (Word variations have been searched)	18,258
#9	(epidemic) (Word variations have been searched)	2966
#10	(epidemiology) (Word variations have been searched)	71,377
#11	(epidemiological investigation) (Word variations have been searched)	15,785
#12	(distribution) (Word variations have been searched)	48,990
#13	(China) (Word variations have been searched)	49,218
#14	#1 OR #2 OR #3 OR #4 OR #5 OR #6 OR #7	7137
#15	#8 OR #9 OR #10 OR #11 OR #12	107,270
#16	#14 AND #15	528
#17	#16 AND 13	34

#### Appendix A.1.4. Search strategies of Embase

**Table A4:** Search strategies of Embase

Search	Query	Items
#1	osteoarthritis OR osteoarthrosis OR “arthritis, degenerative” OR “arthritides, degenerative” OR “degenerative arthritides” OR “degenerative arthritis” OR “osteoarthrosis deformans”	133,383
#2	“prevalence rate” OR epidemic OR epidemiology OR “epidemiological investigation” OR distribution	3,135,066
#3	China	1,880,639
#4	#1 AND #2 AND #3	721
#5	#1 AND #2 AND #3 AND [2000–2018]/py	689

### Appendix A.2. Chinese Database

**Table A5:** Search strategies of four Chinese databases

Database	Query	Items
CBM	(“osteoarthritis” [common field] OR “osteoarthrosis” [common field] OR “arthritis, degenerative” [common field]) AND “distribution” [common field] OR (“survey” [common field] OR “data collection” [common field] OR “epidemic” [common field]) OR “epidemiological” [subject])	310
VIP	U= (“osteoarthritis” OR “osteoarthrosis” OR “arthritis, degenerative”) AND U= (“distribution” OR “survey” OR “data collection” OR “epidemic” OR “epidemiological” OR “prevalence”)	396
CNKI	(KY % ‘osteoarthritis’ + ‘osteoarthrosis’ + ‘arthritis, degenerative’) and (KY % ‘prevalence’ + ‘epidemic survey’ + ‘epidemiological’ + ‘distribution’) not (KY % ‘review’+ ’meta’)	199
Wan Fang	Title or abstract: (“osteoarthritis” or “osteoarthrosis” or “arthritis, degenerate”) and title or abstract: (“prevalence “or “epidemic” or “survey” or “distribution” or “data collection” or “epidemiological”)	567
